# Expectancy Learning from Probabilistic Input by Infants

**DOI:** 10.3389/fpsyg.2012.00610

**Published:** 2013-01-11

**Authors:** Alexa R. Romberg, Jenny R. Saffran

**Affiliations:** ^1^Department of Psychology and Waisman Center, University of Wisconsin – MadisonMadison, WI, USA

**Keywords:** statistical learning, sequence learning, infant, eye-tracking, prediction

## Abstract

Across the first few years of life, infants readily extract many kinds of regularities from their environment, and this ability is thought to be central to development in a number of domains. Numerous studies have documented infants’ ability to recognize deterministic sequential patterns. However, little is known about the processes infants use to build and update representations of structure in time, and how infants represent patterns that are not completely predictable. The present study investigated how infants’ expectations fora simple structure develope over time, and how infants update their representations with new information. We measured 12-month-old infants’ anticipatory eye movements to targets that appeared in one of two possible locations. During the initial phase of the experiment, infants either saw targets that appeared consistently in the same location (Deterministic condition) or probabilistically in either location, with one side more frequent than the other (Probabilistic condition). After this initial divergent experience, both groups saw the same sequence of trials for the rest of the experiment. The results show that infants readily learn from both deterministic and probabilistic input, with infants in both conditions reliably predicting the most likely target location by the end of the experiment. Local context had a large influence on behavior: infants adjusted their predictions to reflect changes in the target location on the previous trial. This flexibility was particularly evident in infants with more variable prior experience (the Probabilistic condition). The results provide some of the first data showing how infants learn in real time.

## Introduction

During their first years of life, infants absorb a staggering amount of information from their environment. Even very young infants are adept at recognizing auditory (e.g., Gervain et al., 2008), visual (e.g., Canfield and Haith, 1991; Kirkham et al., 2002; Bulf et al., 2011), and audiovisual (e.g., Lewkowicz, 2008) patterns, and this structure-extraction ability is thought to play a role in multiple cognitive domains. For example, distributional theories of word segmentation hypothesize that infants track the probability with which syllables follow one another in order to find word boundaries (e.g., Saffran et al., 1996; Aslin et al., 1998) and a similar model has been proposed for segmentation of actions (Baldwin et al., 2008; Roseberry et al., 2011). Word learning is also influenced by distributional information over time; infants can use the co-occurrence of labels and referents across naming events to disambiguate label meanings (Smith and Yu, 2008; Vouloumanos and Werker, 2009).

Over the past two decades, a rich literature has emerged with a focus on the *outcome* of learning, asking whether infants are able to discriminate items consistent with a familiarized structure from those violating that structure. However, few studies have examined how learning unfolds with experience. We do not know how infants’ expectations concerning sequential structure evolve over time, or how they adapt to changes or noise in the structure. Investigations of the learning process are vital to theories of infant knowledge acquisition and development. For example, several studies have compared statistical learning abilities in infants, children, and adults (e.g., Gómez, 2002; Saffran, 2002) and each of these concluded that similar statistical learning skills are available across this developmental span (cf. Bulf et al., 2011; Janacsek et al., 2012). However, the fact that both infants and adults are able to discriminate amongst the same set of test items does not mean that they “solved” the learning problem in the same way. Different groups of participants may have followed different trajectories to similar end points. Crucially, these different trajectories may help explain why infants and adults, with very different brains and experiences, are able to track similar types of structures.

A deeper understanding of the learning process can also contribute to how we conceptualize differences in learning outcomes. A statistically significant difference between group means indicates that infants *can* extract a particular structure, but individual infants may not follow the group pattern. While there are many factors that influence an infant’s behavior on a particular day, a more detailed understanding of the learning process is required to determine whether infants who learned and infants who did not approached the task in the same way.

Recent work in word learning reveals the benefits of investigating learning as an ongoing process. In the cross-situational word learning paradigm, 12- to 14-month-old infants are able to track co-occurrences between labels and objects across trials in order to learn which label reliably co-occurs with each object (Smith and Yu, 2008). However, not all infants learn the label-object mappings equally well. Follow-up experiments using eye-tracking suggested that “learners” exhibited more selective attention during the learning phase (Yu and Smith, 2011) and were better able to resist surface salience to focus visual attention on likely referents (Smith and Yu, 2013). Crucially, these studies go beyond asking whether infants *can* track distributional information to investigate *how* they do it.

Research investigating visual sequence learning provides some important evidence concerning how infants extract structure in time. Infants as young as 2–3 months of age readily form expectations about sequential structure (e.g., Haith et al., 1988; Canfield and Haith, 1991). In these studies, infants viewed images presented sequentially at two (or sometimes three) possible locations. Infants were more likely to make an anticipatory saccade to the location of the next stimulus when the pictures followed a regular spatiotemporal sequence than when they were randomly presented, providing evidence of sensitivity to sequential structure (Haith et al., 1988; Canfield and Haith, 1991; Canfield and Smith, 1996; Wentworth et al., 2002). These results suggest that anticipating future events is an important aspect of sequence learning in infancy.

In fact, anticipation plays a central role in many models of learning (Bar, 2007). Simple recurrent network models (SRNs) learn sequential structure specifically by making a prediction about the next element in a sequence and then using the actual input as the teaching signal to revise future expectations (Elman, 1990). The dopamine system in the brain codes the difference between expected and actual outcomes (e.g., Schultz, 2002), providing a neural substrate for learning from prediction. The perceptual learning process of using transitional probabilities to segment words from speech is often described as shaping the learner’s expectancies of upcoming input (e.g., Gomez et al., 2011). Thus, measuring infants’ expectations will be important for understanding the learning process.

The current study focused on the process of expectancy learning in order to address several important questions that have not previously been the focus of investigation in the infant learning literature. The first is the form of infants’ expectations for noisy, or probabilistic, information. The second is how infants update their expectations when they encounter new information. The nature of infants’ representations of noisy input has important implications for how we conceptualize learning during the first few years of life. Developmental theories that rely on infants accruing information over time, such as statistical language learning (see Romberg and Saffran, 2010, for review) or other association-based accounts (e.g., Smith, 2000) must address the fact that few relationships in the real world are deterministic. However, studies of sequence learning in infancy have so far primarily tested infants’ recognition of perfectly predictable transitions.

Outside of infancy research, the study of learning from probabilistic information has a long history. Probability learning was extensively studied in the 1950s and 1960s, and that work continues today in the field of decision-making. One frequently used paradigm involves asking participants to predict which of two outcomes will occur on a given trial (e.g., which of two lights will turn on). Researchers have explored different models of learning employed by adults and children (for review see Estes, 1972; Hogarth, 1975). This literature reveals that participants’ expectations for binary outcomes are driven by a large number of factors, including the overall probability of a particular outcome, the local sequential context (e.g., the length of a run of a particular outcome), and response biases (e.g., the gambler’s fallacy or an expectation of alternation). Participants’ responses are strongly influenced by the specifics of the task, and children of different ages tend to respond in different ways (e.g., Erev and Barron, 2005; see Bogartz, 1965 for an example in preschool children).

Several studies have found that infants’ attend more to sequences containing a “medium” level of information, relative to very predictable or very unpredictable sequences (Haith et al., 1969; Collins et al., 1972; Kidd et al., 2012). However, we do not know how predictable a sequence must be to support learning, or how sensitive infants’ expectations are to the information content of a sequence. In one recent study, 10-month-old infants exposed to a sequence in which the target appeared at the same location on 70% of trials failed to anticipate the target at the more likely location (Davis et al., 2011), suggesting that events must be highly regular to elicit specific expectations.

One set of studies that may inform our thinking about how infants handle probabilistic information examined young infants’ expectations for asymmetrical sequences (Canfield and Haith, 1991; Canfield and Smith, 1996). In these studies, 2- to 5-month-old infants observed sequences of pictures that appeared on either the right or left side of a screen. The pictures either followed a regular asymmetrical pattern of L-L-R or L-L-L-R, a regular symmetrical pattern of L-R-L-R, or an irregular pattern in which the position of the picture was unpredictable. In these asymmetrical sequences, some individual transitions are probabilistic (i.e., a left picture can be followed by another picture on the left or a picture on the right) but all transitions are deterministic if one is able to keep track of where one is in the sequence (i.e., viewing the first or second left picture). Infants increased their anticipations for pictures on the right side following the last left-side picture relative to the first left-side picture, suggesting that they were able to use the sequential context to partially resolve the ambiguity of individual transitions. Interestingly, though, after a right side picture infants did not reliably anticipate the return to the left-side despite the perfect reliability of that transition. It is unclear if this failure is due to the structure of the sequence itself or because young infants find it challenging to reverse the direction of their eye movements (Wentworth and Haith, 1998). Similar inconsistencies have been found in other studies of sequence learning with 3-month-olds (Wentworth et al., 2002), suggesting that a deeper look at infants’ expectancy learning is required.

In a separate study, 3-month-old infants each viewed a regular alternating sequence and an irregular (unpredictable) sequence, with six infants viewing the regular sequence first and six viewing the irregular sequence first (Haith et al., 1988). The two groups showed the same amount of facilitation when reacting to the appearance of a picture in the regular sequence compared with the irregular sequence. However, infants who viewed the irregular sequence first showed a larger increase in anticipations for the regular sequence than the other group. The authors interpreted this difference as indicating that infants needed some time to settle in to the experimental paradigm. However, another possibility is that the initial exposure to the irregular sequence improved learning of the alternating sequence. This question will be directly addressed in the current experiment.

In the present study, we investigated the process underlying infants’ expectancy learning and compared learning trajectories for sequences that varied in their degree of noise (probabilistic vs. deterministic structure). Because we intended to interrogate learning as it happened, we used a task that infants should easily learn. We measured infants’ gaze as they viewed sequences of a central fixation cue followed by a peripheral target on either the left or right side of the screen (Johnson et al., 1991). During the initial phase of the experiment, infants either saw targets that appeared consistently in a single location (Deterministic condition) or probabilistically in two possible locations, with one side three times more frequent than the other (Probabilistic condition). After this initial divergent experience, both groups observed the same sequence of trials for the rest of the experiment. The target appeared at the same location for all of these trials, with the exception of two “low-probability (LP)” sets (described below). Broadly, the current study asked how infants’ expectations develop over time as they gather evidence pointing to the most likely target location. We compare infants’ behavior between the two conditions to determine how the predictability of the initial input influences predictions as the experiment progresses. We first examined learning across the experiment as a whole and then focused on specific sets of trials to determine how infants update their expectations from one trial to the next.

Participants in the current study were 12-month-old infants. Pilot testing revealed that infants of this age made anticipatory eye movements on most trials in paradigms similar to the one used, allowing for data of sufficient density to investigate how infants’ anticipations changed over multiple timescales within the experiment. We were also interested in focusing on 12-month-olds because they are at an ideal age for future extensions of these learning questions into other domains: they show sensitivity to social cues (e.g., following adults’ gaze; Woodward, 2003) and have begun to build a receptive vocabulary. As noted above, 12-month-olds can successfully track label-object associations across ambiguous contexts (Smith and Yu, 2008), raising interesting questions about how infants represent and update these associations.

In the first set of analyses, we explored the time course of learning from probabilistic input. We asked whether infants in the Probabilistic condition were able to detect where the target was most likely to appear, whether they predicted the most likely target location at the same rate as infants in the Deterministic condition, and whether the early variability in target location continued to influence infants expectations late in the experiment. The consistent target location in the Deterministic condition should enable infants to anticipate the target’s appearance; research using a similar paradigm revealed that infants can make anticipatory saccades to a consistent target location within the first 10 trials (Kovács and Mehler, 2009). Whether infants in the Probabilistic condition would also predict the most likely target location was less clear. It seems reasonable to expect that the inconsistent input would lead them to take more trials to begin preferentially predicting the higher probability target location. However, it is also possible that they will not learn to anticipate the more likely target location (see Davis et al., 2011). Finally, infants in the Probabilistic condition may predict the likely target location at a rate similar or equal to the infants in the Deterministic condition, neglecting the “noise” in the target location to maximize time spent viewing the target.

In the second set of analyses, we examined the dynamics of infants’ gaze within specific trials to see how their expectations are shaped by the local and global context. Specifically, we focused on two “LP” sets of trials to ask whether a change in target location has an immediate effect on infants’ predictions and whether that effect is moderated by the target’s prior predictability. On the first trial in each set the target appeared in the less likely location, and on the second trial in the set the target returned to the higher probability location. By comparing infants’ anticipatory looking across the two trials in each set, we can measure how infants’ expectations were influenced by the change in target location.

There are several possible outcomes for this analysis. A single novel instance may not affect the predictions of infants who have seen the target appear at one location very consistently in the past; they may continue to predict the most likely target location at the same rate, effectively treating the change as a random blip. Infants who have seen the target appear in multiple locations, however, may more readily shift their predictions from one trial to the next. This account would predict that infants in the Probabilistic condition should adjust their behavior more than the infants in the Deterministic condition during the “LP” set.

However given that error signals convey the difference between expected and actual outcomes (e.g., Elman, 1990; Schultz, 2002), infants with stronger expectations for the target location may get a stronger feedback signal to revise their behavior when those expectations are violated. If that feedback drives immediate changes in behavior, infants for whom the change in target location is more surprising (those with deterministic prior experience) would change their behavior more than those for whom it is less surprising (those with probabilistic prior experience, who have seen the target in both locations). Similarly, the Rescorla–Wagner model predicts that the amount of learning on a given trial is proportional to the difference between the current association strength between the cue and target location and the maximum possible association. Finally, it is possible that infants in both conditions will actually strengthen their expectation for the target in the more likely location after the change in target location because no infants had ever seen multiple targets in a row at the lower-probability location^1^. In order to test these contrasting predictions, we used growth curve models to explore the gaze dynamics of individual trials and provide a detailed picture of how infants’ expectations are shaped by their ongoing experiences. We also compared behavior on the first and second LP trial set to examine the effects of accumulated experience.

## Materials and Methods

### Participants

Forty-eight infants aged 11.5–13.5-months (range 348–409 days, *M* = 377, SD = 18.3) were randomly assigned to one of two conditions (Deterministic or Probabilistic). There were no significant differences between conditions on age or gender. Nine additional infants were excluded from the final analyses due to fussiness or inattention during the study (7), or failure to make anticipatory eye movements on more than 25% of trials (2). Informed consent for each infant participant was obtained from a legal guardian and study procedures were approved by the Social and Behavioral Science Institutional Review Board at the University of Wisconsin – Madison.

### Materials

**Figure 1:**
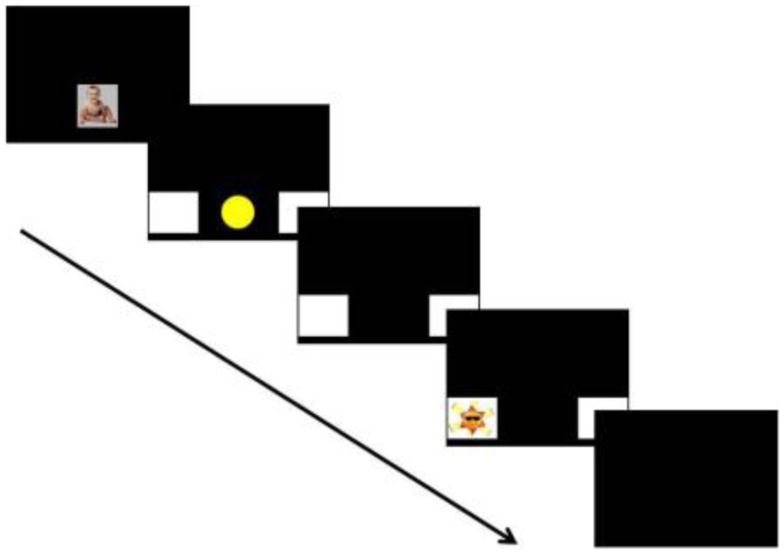
**The structure of an individual trial**. See text for the duration of each slide.

Each trial began with an attention-getting stimulus (a smiling baby accompanied by music) that was displayed until the infant was facing forward and attending. This was followed by a fixation cue: a yellow circle that loomed in the center of the screen and played a xylophone sound effect. Two white boxes flanked the circle to the left and right. The circle cue played for 1500 ms and then disappeared; the white boxes remained on screen. After an 800 ms delay to allow infants to launch a saccade, the target appeared in one of the boxes. The target consisted of a 2000 ms animated toy with music, with one of five different animations presented on each trial. Trials ended with 500 ms of silence and a blank screen. All stimuli were approximately 12″ square and horizontally aligned at or just above the infants’ eye height. The peripheral stimuli were positioned with their centers 20″ from the center of the screen. A schematic of the trial structure is shown in Figure 1.

### Procedure

Infants were seated on their caregiver’s lap in a dimly lit sound-attenuated booth, approximately 36” from a large projection screen. A short (10 s) animation was used to orient infants to the screen. This was followed by 21 trials for a total study length of approximately 2.5 min. Caregivers wore opaque glasses so they could not see the stimuli.

For each infant, the target appeared in one peripheral location for the majority of trials [the *high probability* (HP) *side*] and in the other peripheral location (the LP *side*) on the remaining trials. The location of the HP side was counterbalanced across participants for both conditions. The full trial sequence is given in Table 1.

**Table 1:** **Target location for each trial (HP, High Probability side; LP, Low-Probability side)**.

Block	Trial	Deterministic condition		Probabilistic condition	
		Target location	*P*(HP)	Target location	*P*(HP)
−	1	HP	−	HP	−
1	2	HP	1	LP	1
	3	HP	1	HP	0.5
	4	HP	1	HP	0.67
	5	HP	1	HP	0.75
2	6	HP	1	LP	0.80
	7	HP	1	HP	0.67
	8	HP	1	HP	0.71
	9	LP	1	LP	0.75
3	10	HP	0.89	HP	0.67
	11	HP	0.90	HP	0.70
	12	HP	0.91	HP	0.73
	13	LP	0.92	LP	0.75
4	14	HP	0.85	HP	0.69
	15	HP	0.86	HP	0.71
	16	HP	0.87	HP	0.73
	17	HP	0.88	HP	0.75
5	18	HP	0.88	HP	0.77
	19	HP	0.89	HP	0.78
	20	HP	0.90	HP	0.79
	21	HP	0.90	HP	0.80

**P*(HP) is the probability of the target appearing on the HP side based all previous trials within that condition*.

Infants were randomly assigned to one of two conditions that differed only on the target location for the first six trials. In the Deterministic condition, the target appeared on the HP side for all six of these trials. In the Probabilistic condition, the target appeared on the HP side for four of the first six trials, and on the LP side for two of the trials (trials 2 and 6). The location of the target could not be perfectly predicted in this condition. Beginning with trial 7, both conditions followed the same sequence of target locations. The two “LP” sets spanned Trials 9–10 and 13–14 for both conditions. The target appeared on the LP side on trials 9 and 13 and on the HP side for trials 10 and 14. The target appeared on the HP side for all other trials for both conditions.

A camera mounted below the screen recorded a close-up of the infant’s face, sampling the infant’s gaze at 30 Hz. Trained coders blind to the target location stepped through the videos one frame at a time. Infant looks were coded as left box, right box, center, shifting between locations, or off task. A second coder recoded 10% of trials and any discrepancies were resolved through discussion.

On each trial, there were three possible outcomes: an anticipatory shift to the HP target location, an anticipatory shift to the LP target location, or a reactive shift once the target appeared. The anticipatory window began at trial onset and ended 200 ms after target onset (2500 ms into the trial). We used 200 ms after target onset as a cut-off because that is approximately the time necessary to plan an eye movement (Canfield et al., 1997; Reznick et al., 2000); the pattern of results is unchanged if a cut-off of 133 or 167 ms is used instead.

## Results

### Data quality

Overall, infants were very attentive. The first trial was not analyzed, as infants did not yet have any information on which to base an anticipatory eye movement. Trials that were deemed invalid by the coder were excluded, including those on which the infant was fussy or inattentive for more than 1000 ms of the 2500 ms anticipatory window, the infant’s eyes were not visible, or the parent interfered by talking or pointing to the screen. Of 960 possible total trials, 46 were excluded (4.8%). There was no difference between conditions on the number of invalid trials excluded (Deterministic = 22, Probabilistic = 24).

Infants made anticipatory shifts on the majority of trials and there was no difference between conditions in the proportion of trials with an anticipatory shift [Deterministic *M* = 0.84, SD = 0.16; Probabilistic *M* = 0.80, SD = 0.23; *t*(46) < 1]. This information suggests that infants were equally engaged in the task regardless of experimental condition.

### Infants’ predictions across the experiment

These analyses focus on whether infants in the Probabilistic condition anticipated the target at the HP location more often than chance and whether anticipatory behavior is the same across the Deterministic and Probabilistic conditions. As Table 1 shows, the cumulative probability of the target appearance in the HP location differs by about 33% between the conditions after the initial six trials. The groups’ experiences overlap more as the study continues, and by the end of the study, the respective cumulative probabilities come close to converging. Thus, comparing performance early in the experiment allows us to determine the sensitivity of infants to that difference in predictability, while comparing performance at the end of the experiment allows us to determine whether the early variability in the Probabilistic condition continues to influence infants’ expectations even as the materials become less variable.

**Figure 2:**
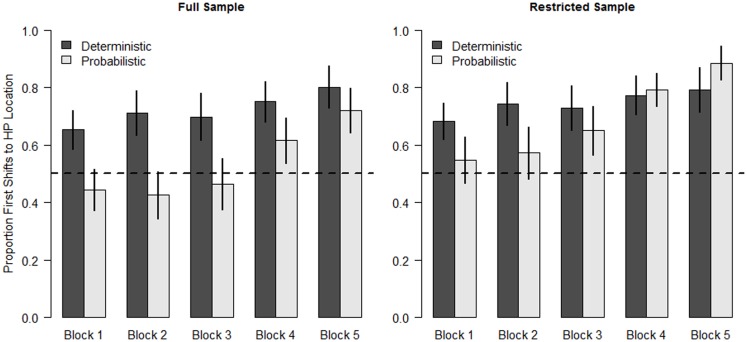
**Proportion of anticipatory shifts to the High Probability location in each condition**. Error bars are standard error of the mean. The left panel shows results from all infants, the right panel includes only the restricted sample (see text).

To investigate infants’ expectations over time, we divided the experiment into blocks of four trials (see Table 1). We analyzed the first anticipatory shift, defined as the first location to which infants shifted from the central fixation cue during the anticipatory window. The mean proportion of anticipatory first shifts to the HP location for each block is shown in Figure 2. This proportion was calculated as the number of first shifts to the HP location divided by the number of anticipatory shifts. The pattern of results is unchanged when the total number of trials is used as the denominator instead. Inspection of the graph reveals that both groups increased their proportion of anticipatory eye movements to the HP location over the course of the experiment. As expected, the Deterministic group began to predict the reward location earlier in the study than the Probabilistic group, with markedly more anticipation of the HP location in the early blocks.

To statistically test the patterns of infants’ anticipatory behavior, we fit the first anticipatory shift data with logistic mixed-effects models. Trials with a first anticipatory shift to the HP location were scored with a 1 and those with a shift to the LP location were scored with a 0. Trials with no anticipatory shift were excluded from this analysis. Because infants made anticipatory shifts on the vast majority of trials, the pattern of results is the same if all trials are included. However, including only trials with an anticipatory shift allows us to ask specifically whether infants anticipated the target more frequently at one location than the other by comparing accuracy to a chance-level of 50%. Models were fit using the lme4 package in R with the binomial link function (Bates and Maechler, 2010; R Development Core Team, 2010). The verbose command was used to check for model convergence.

First we asked whether infants learned where the target was more likely to appear. For each block, data for each condition were separately fit with a model that included random effects for subject and the intercept as the only fixed effect. A significant positive intercept indicates that infants were more likely to shift to the HP location than the LP location in that Block while intercepts of zero indicate that infants shifted with equal probability to the HP and LP locations. Results are given in Table 2. The consistent early target location led to rapid and reliable anticipation of the target at the HP location in the Deterministic condition: Infants in this condition had significant positive intercepts for all five blocks. In contrast, infants in the Probabilistic condition only exceeded chance performance in the fifth and final block. This pattern of results suggests that infants in the Probabilistic condition did not form stable expectations for the target location until late in the experiment, after they had experienced a run of at least four targets in the HP location.

**Table 2:** **Results from intercept models for each experimental block testing whether infants anticipated the target more frequently at the HP location than expected by chance**.

Condition		Block 1	Block 2	Block 3	Block 4	Block 5
Deterministic	*b*	0.61	2.00	2.56	1.72	5.43
	*z*	1.97	2.98	2.85	3.41	3.23
	*p*	0.05	0.003	0.004	<0.001	0.001
Probabilistic	*b*	−0.48	−0.53	−0.38	0.53	1.91
	*z*	1.44	1.17	0.78	1.21	2.75
	*p*	0.15	0.24	0.44	0.23	0.006
Probabilistic restricted sample	*b*	0.02	0.29	0.48	1.20	2.49
	*z*	0.06	0.67	1.41	3.66	4.40
	*p*	0.96	0.50	0.16	<0.001	<0.001

Next, we asked whether the differences between conditions were significant. We fit the first anticipatory shift data with a mixed-effect logistic model with Condition (Deterministic vs. Probabilistic) as a between-subjects factor and Block (1–5) as a within-subjects factor. Condition was contrast coded for main effects and Block was centered. Uncorrelated random effects of Subject on the intercept and Block were included (correlating the random effects did not improve model fit). The fixed effects were uncorrelated (highest *r* = 0.041).

The models confirmed that anticipatory shifts to the HP location increased across blocks for both groups, and that infants in the Deterministic condition made more first shifts to the HP location than infants in the Probabilistic condition. The main effect of Block was significant (*b* = 0.39, *z* = 4.20, *p* < 0.001). The main effect of Condition (*b* = 1.48, *z* = 2.70, *p* = 0.007) indicated that the odds of a first anticipatory shift to the HP location were 4.4 times greater for infants in the Deterministic condition than for infants in the Probabilistic condition. The interaction between Block and Condition was not significant (*p* = 0.61).

The regression model reveals that, consistent with learning the sequence, infants in both conditions increased their rate of anticipatory looking to the HP location over time. In addition, the consistency of the sequence during the first six trials of the experiment influenced how readily infants anticipated the HP location: infants in the Probabilistic condition made fewer anticipatory shifts to the HP location. The fact that Block did not interact with Condition indicates that differences between the two conditions were maintained throughout the experiment. The lack of interaction, combined with the chance-level performance in the Probabilistic condition until Block 5, suggests that the early variability in target location continued to influence infants’ predictions late in the experiment, despite the fact that infants in both conditions received the same experience from Trial 7 on.

**Figure 3:**
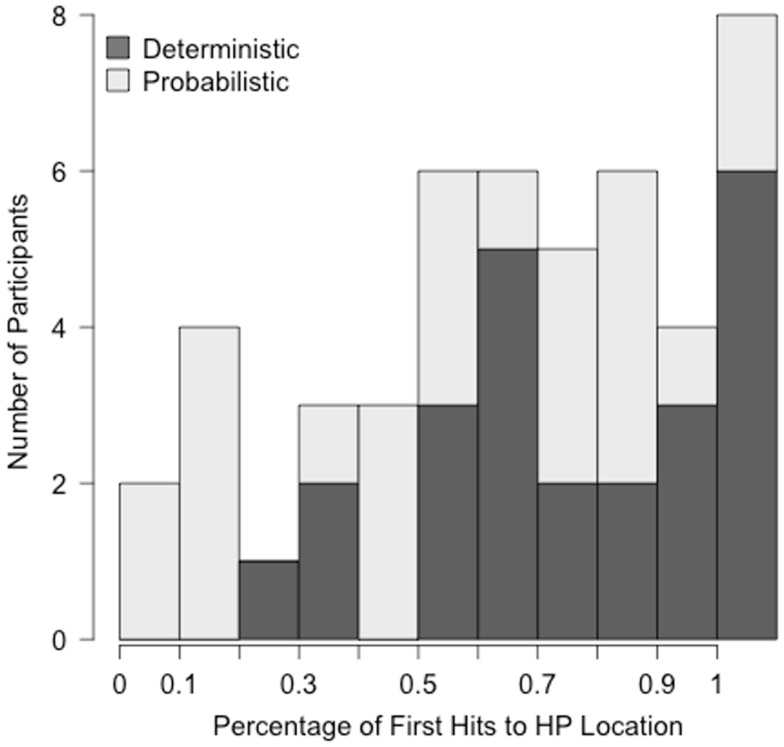
**Frequency histogram displaying the rate at which infants anticipated the HP location across both conditions (excluding Trial 1)**.

The lack of a significant Block by Condition interaction in the regression model is somewhat surprising given the pattern of means in Figure 2, in which the difference between the two groups is quite a bit smaller in Block 5 than Block 1. This raises the question of how well the group means represent the individual data, as our ability to detect an interaction may have been hampered by a large amount of individual variability. Indeed, infants responded to the materials in different ways. Qualitatively, about half of all infants (10 in the Deterministic and 15 in the Probabilistic condition) followed a pattern similar to the group means, with increasing first shifts to the HP location across blocks. Some infants, however, showed no change in their predictions and consistently anticipated the target in the same location throughout the experiment. The overall rates of first anticipatory shift to the HP location for individual infants are shown as a histogram in Figure 3.

The grand mean of the proportion of first shifts to the HP location was 64.9%, with a standard deviation of 29.2%. Infants whose performance was at least one standard deviation above the mean predicted the HP location on a minimum of 94% of anticipatory shifts. There were 10 infants who fell into this category, eight in the Deterministic and two in the Probabilistic condition. Infants whose performance was at least one standard deviation below the mean predicted the HP location less than 35% of the time. There are seven infants who fell into this category, one in the Deterministic and six in the Probabilistic condition. In fact, these infants predicted the HP side much less than 35% of the time: the infant from the Deterministic condition did soon 25% of anticipatory shifts and those from the Probabilistic condition did so on less than 17%. The consistency in predictions (HP or LP) was not a byproduct of a low rate of anticipations: Infants at the high and low ends of the distribution made anticipatory shifts on the same number of trials as those close to the mean (18.5 and 19.3, respectively, Wilcoxon rank sum test *W* = 210, *p* = 0.248).

Consistently predicting the target in the HP location aligns with optimal behavior on this task (maximizing time spent viewing the target animation). Consistently predicting the LP location, however, is more difficult to interpret. There are a number of explanations for the behavior of this subset of the sample that our data do not allow us to distinguish. One possibility is that these infants are incorrectly representing the probability that the target will appear on each side of the screen. This explanation suggests a problem with their ability to detect regularities. A second possibility is that these infants learned that if they shift their eyes to one location, the target appears in the opposite location (and they can then reactively shift to look at it). They may be correctly representing the probabilities but the behavior that has been reinforced is the shift to the LP location. Because the target appearance was not contingent on the infants’ behavior, it is possible that some infants could associate a shift to the LP location with the appearance of the target in the other location – what Skinner has called “superstitious behavior” (Skinner, 1948). Another possibility is that these infants have a bias in their eye movements that makes them prefer a first shift to one location, and their eye movements do not reflect their knowledge of the sequence. (Stimuli were counterbalanced so that for half of the infants the HP side was the right and the other half the left, but this does not prevent individual infants’ biases from influencing their behavior). This explanation seems unlikely, however, as it does not account for why infants in the Probabilistic condition were more likely to exclusively predict the LP than the HP locations.

Indeed, nearly all of the infants who consistently predicted the LP location were in the Probabilistic condition. This pattern raises the possibility that the LP events on trials 2 and 6 led some infants to preferentially predict the LP location. The greater variability in the target location for the Probabilistic condition appears to have rendered greater variability in behavior. This finding is interesting in itself and potentially represents an important property of learning from probabilistic systems. The LP-predicting infants behave (apparently) systematically, but their behavior is impossible to interpret in the current context. In the analysis above we interpreted the main effect of Condition and lack of a Condition by Block interaction to mean that the early LP trials had a lasting effect on infants in the Probabilistic condition, continuing to influence their expectations at the very end of the experiment. Because the LP-predicting infants make up 25% of the sample in the Probabilistic condition, their behavior may account for a large portion of the difference between the conditions. This raises the question of whether we would come to the same conclusions about the influence of probabilistic information if we restrict the sample to those infants whose behavior is interpretable.

In order to get as complete a picture as possible of how infants learn from probabilistic information, we examined the data excluding the 7 LP-predicting infants (six from the Probabilistic condition and 1 from the Deterministic condition). The means for the remaining infants are shown in the right panel of Figure 2. The regression models testing Condition and Block revealed a highly significant main effect of Block (*b* = 0.39, *z* = 4.67, *p* < 0.001). However, in contrast to the model with the full sample, the main effect of Condition was not significant (*b* = 0.53, *z* = 1.15, *p* = 0.249), indicating that, overall, infants in the two conditions anticipated the target at the HP location at similar rates. The Condition by Block interaction was not significant. Thus, the restricted sample of infants in the Probabilistic condition did not show long-lasting effects from the early target variability. Intercept models fit to each block of data for the Probabilistic condition reveal that this restricted sample of infants reliably anticipated the target at the HP location in Blocks 4 and 5 (see Table 2, intercept models for only the Probabilistic condition were re-run because the means for the Deterministic condition were virtually unchanged). In fact, in Block 5, infants in the Probabilistic condition made numerically more anticipatory shifts to the HP location than the infants in the Deterministic condition. The different results for full and restricted samples suggests that while the early inconsistency in the target location had lasting effects for the group as a whole, this was not true for all infants. This point will be returned to in the Discussion.

When testing young infants, Haith et al. (1988) found that pre-exposure to an irregular sequence may have facilitated anticipatory eye movements during a regular sequence. The last eight trials of our experiment were all HP location trials. Thus, it is possible to view the first 13 trials in the Probabilistic condition as a pre-exposure to an irregular pattern, and to compare infants’ predictions for the *last* two blocks of the experiment with the predictions of infants in the Deterministic condition for the *first* two blocks (i.e., a deterministic target location without pre-exposure). A logistic mixed-effects model of these data with Condition as a fixed effect and Subject as a random effect did not find an effect of Condition for either the full or restricted samples (all *p* > 0.15). Thus, we did not find that the early variability in target location in the Probabilistic condition facilitated anticipations once the target location became regular.

### Infants’ trial-by-trial updating

One of the primary questions motivating this study was how infants incorporate new information into their expectations. In this analysis, we asked whether infants altered their expectations after they see the target appear in an unlikely location. We looked in detail at infants’ gaze on the two “LP” sets that spanned Trials 9–10 and 13–14. The target appeared in the LP location for all infants, on Trials 9 and 13. The study was designed to examine the effect of unexpected events as a function of how novel they are. Prior to Trial 9, infants in the Deterministic condition had never seen the target appear in the LP location, while infants in the Probabilistic condition had seen the target appear in the LP location twice (on Trials 2 and 6). Thus, at Trial 9 infants in the Deterministic condition should have a stronger expectation that the target will continue to appear on the HP side than infants in the Probabilistic condition. This is corroborated by the Block means shown above, in which infants in the Deterministic condition had a higher rate of first shifts to the HP location during Block 2.

Infants in the Deterministic condition should be relatively more surprised than infants in the Probabilistic condition at the appearance of the target in the LP location, leading to the generation of a larger error signal. If the size of this error signal drives immediate changes in behavior, we would expect to see a larger trial-by-trial change in the Deterministic than the Probabilistic condition for the trials just after these unexpected events (Trials 10 and 14). It is also possible, though, that the highly consistent prior experience by infants in the deterministic condition may buffer infants from small inconsistencies in the local context. If that is the case, we would expect the less consistent prior experience in the Probabilistic condition to drive larger trial-by-trial changes in behavior.

Finally, it is also possible that we would see a different pattern of results for the earlier LP trial set (9–10) vs. the later set (13–14) due to the accumulation of experience across the study. In particular, the two groups have had more experience in common by Trial 13 than they do by Trial 9. Therefore, the expectations generated by infants in the two groups may be more similar for the second trial pair, leading to more similar adjustments in behavior on Trial 14 than on Trial 10.

**Figure 4:**
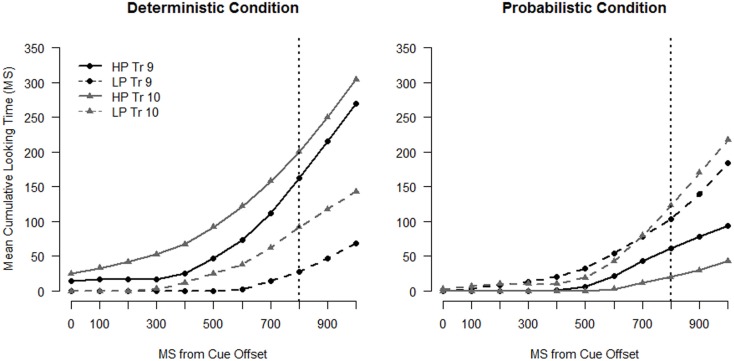
**Cumulative time spent looking to the high probability (HP) and low-probability (LP) location for infants in the Deterministic condition (left panel) and infants in the Probabilistic condition (right panel) for Trial 9 and Trial 10**. The dotted vertical line indicates target onset.

For the trial-by-trial analyses, we employed growth curve models of infants’ looking behavior. Recent work from a number of labs has demonstrated that growth curve analysis provides a useful model of the dynamics of looking behavior within and across trials (e.g., Barr, 2008; Mirman et al., 2008; Mirman and Magnuson, 2009; Trueswell and Papafragou, 2010; Barr et al., 2011). These models take advantage of the richness of eye-tracking data by testing the shape of the curves formed by plotting looking to the areas of interest against time (see Figure 4) and provide more power than methods that reduce several seconds of looking behavior to a binary outcome.

We followed Trueswell and Papafragou (2010) in using the cumulative sum of looks to the two possible target locations as our dependent measure. The cumulative sum of time spent on the HP and LP locations tells us how infants’ preference for the HP location develops as the trial unfolds. The cumulative sum profiles averaged across subjects for the first “LP” set are shown in Figure 4 with separate curves for the HP and the LP locations. The plots depict looking during the anticipation window. Each point of the plot represents the mean number of frames that infants have spent looking at that location up to that point in the trial. Accelerating slopes indicate that infants are switching onto that location and decelerating slopes indicate that infants are switching off of that location. The data were down-sampled to 10 Hz (as shown in the plots) in order to correct for the non-independence of eye movements in the analyses that follow (see Barr, 2008).

**Figure 5:**
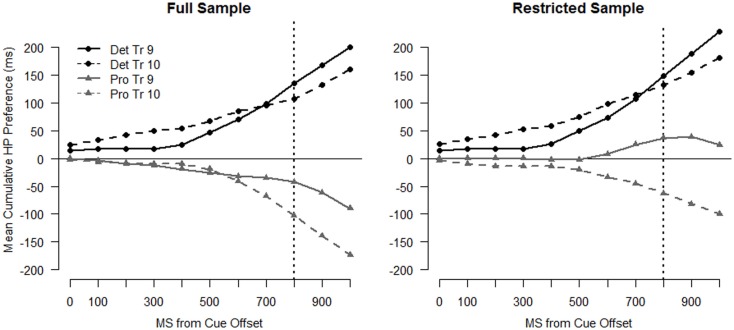
**Cumulative preference profiles for Trials 9 and 10**. Points above 0 indicate a preference for the HP location and points below 0 indicate a preference for the LP location. The left panel depicts data from the full sample and the right panel depicts data from the restricted sample (see text). The dotted vertical line indicates target onset.

To simplify the analyses, rather than model looking time to each location separately we modeled the cumulative *preference* for the HP location (see Trueswell and Papafragou, 2010). These curves are shown in the left panel of Figure 5. Values were calculated by subtracting the cumulative sum of looking time to the LP location from looking time to the HP location for each sample. Therefore, these preference curves indicate how much *more* looking time has been accumulated to the HP than the LP location at each sample. A positive slope indicates that infants are accumulating looking time to the HP location more rapidly than the LP location at that point in the trial and a negative slope indicates that infants are accumulating looking time to the LP location more rapidly than the HP location.

If infants anticipate the target at the HP location, the preference curves will be above 0 and have positive slope. If the appearance of the target in the LP location causes a shift in infants’ predictions in favor of the LP location, the preference curve on the subsequent trial will be less positive overall and/or have a less positive slope (a curve that falls below zero would indicate overall preference for the LP side). In the following analyses, we tested differences in preference curves within the two LP trial sets: Trial 9–10, and Trial 13–14. For each analysis, we looked first at the entire sample and then followed up with an analysis on the restricted sample that excludes the seven LP-predicting infants identified above. The full sample analysis provides a description of how infants as a whole adjusted their expectations after the LP target appearance. The analysis on the restricted sample focuses on those infants who demonstrated sensitivity to the probability structure. By necessity, this analysis has unequal group size due to the different numbers of LP-predicting infants in each group (see model results in the Appendix for exact sample size for each analysis).

We fit the preference curves with growth curve models using the lme4 package in R (Bates and Maechler, 2010). The shape of the curves was modeled with orthogonal polynomials (linear and quadratic terms) and fixed effects were entered for Condition, Trial and all interactions. Random effects of Subject on Intercept were included, as well as interactions between Subject and Linear Time, Subject and Quadratic Time, and Subject and Trial. A positive linear slope indicates that infants’ preference for the HP location increases over the course of the anticipation window and a positive quadratic slope indicates greater gains for the HP location toward the end of the anticipation window. Because of the challenges in determining degrees of freedom for mixed-effects models, the lme4 package does not provide *p*-values for the *t* statistics (for discussion, see Baayen et al., 2008). The significance of the interaction terms was determined using a log-likelihood ratio test between models including and excluding the interaction terms.

## First Low-Probability Set: Trials 9 and Trial 10

The cumulative sum profiles for Trials 9 and 10 (Figure 4), and the corresponding preference curves (Figure 5, left panel) for the Deterministic condition, are as expected for infants anticipating the HP target location. The curve for the HP location is higher (shifted up) and steeper than the curve for the LP location for both trials, indicating that infants accumulated looking time more rapidly to the HP location than to the LP location. The profiles for the full sample of the Probabilistic condition (Figure 4, right panel and Figure 5), on the other hand, show a preference for the LP location overall for both Trials 9 and 10.

Results from the statistical model on the entire sample, shown in Table A1 in Appendix, support the hypothesis that infants adjusted their expectations after the LP target event on Trial 9, preferring the HP side less on Trial 10 than Trial 9. However, there was not a difference in the extent of change between the two conditions. There were significant two-way interactions between Trial and Linear Time [χ^2^(1) = 29.25, *p* < 0.001] and Trial and Quadratic Time [χ^2^(1) = 7.04, *p* = 0.008], indicating that the Trial 10 curves are shallower and have less positive slope than the Trial 9 curves. The three-way interactions between Condition, Trial, and Linear (and Quadratic) Time were not significant (*p* > 0.3), nor was the two-way interaction between Trial and Condition [χ^2^(1) = 0.397, *p* = 0.529], indicating that the strength of the effects of Trial on the preference for the HP location was similar across conditions.

The model also confirmed that infants in the Deterministic condition had stronger expectations that the target would appear on the HP side. There were significant two-way interactions between Condition and Linear Time [χ^2^(1) = 9.16, *p* = 0.002] and Condition and Quadratic Time [χ^2^(1) = 4.54, *p* = 0.033], as well as a significant main effect of Condition (*t* = 3.505). These effects indicate that preference curves for the Deterministic condition were more positive overall, had more positive slope, and had more positive acceleration than those for the Probabilistic condition.

For the sample as a whole, the two groups of infants exhibited different expectations going into Trial 9. Infants in the Deterministic condition anticipated the target in the HP location, while those in the Probabilistic condition actually showed a slight preference for the LP location. While the dynamics of infants’ gaze across the two groups were fairly different (see Figures 4 and 5), the net change in preference for the HP location from Trial 9 to Trial 10 was similar across the groups, suggesting that the strength of infants’ expectations going into the “LP” trial set did not moderate infants’ response to the change in target location. However, on Trial 10 infants in the Deterministic condition maintained their overall preference for the HP location, while infants in the Probabilistic condition more strongly preferred the LP location. We next asked whether the differences between the conditions seen in the trial-by-trial analysis was caused by the subgroup of LP-predicting infants.

### Analysis excluding LP-predicting infants

Interestingly, the differences between the conditions described above for the full sample are maintained and even amplified within the smaller sample. The preference curves for Trials 9 and 10 for the restricted sample are shown in the right panel of Figure 5 and the full growth curve model results are given in Table A2 in Appendix. In addition to the overall stronger preference for the HP location in the Deterministic condition, a significant interaction between Condition, Trial, and Linear Time [χ^2^(1) = 5.12, *p* = 0.03] indicates that when considering only the restricted sample, the LP target placement on Trial 9 had a larger influence on infants in the Probabilistic condition than infants in the Deterministic condition.

To better understand the interaction, individual models were fit for each condition. The model for the Deterministic condition revealed that infants accumulated looking time to the HP and LP sides at a more even rate on Trial 10 than they did in Trial 9. There were significant interactions for Trial with both Linear and Quadratic Time, indicating that the preference slope for Trial 10 was shallower than that for Trial and did not accelerate during the anticipation window [Trial by Linear Time: *b* = −1.96, SE = 0.68, log-likelihood ratio test: χ^2^(1) = 8.16, *p* = 0.004; Trial by Quadratic Time: *b* = −1.69, SE = 0.68, χ^2^(1) = 6.06, *p* = 0.014]. Inspection of Figure 5 makes clear that infants still prefer the HP side overall, as does the positive main effect of Linear and the positive intercept in the model (Linear Time: *b* = 5.81, SE = 2.10, *t* = 2.76; intercept: *b* = 2.54, SE = 0.76, *t* = 3.37). In fact, it appears that infants in the Deterministic condition increased their attention somewhat to *both* locations on Trial 10 relative to Trial 9, but the net result was a decrease in preference for the HP location.

The model for the Probabilistic conditional also revealed a decrement in preference for the HP side on Trial 10 relative to Trial 9. Infants in the Probabilistic condition actually accumulated looks to the LP location at a higher rate than looks to the HP location on Trial 10, as indicated by significant interactions between Trial and both Linear and Quadratic Time with no other effects with *t*-values > 2 [Trial by Linear Time *b* = −4.14, SE = 0.70, χ^2^(1) = 33.13, *p* < 0.001; Trial by Quadratic Time *b* = −1.46, SE = 0.70, χ^2^(1) = 4.34, *p* = 0.037]. Infants also showed no preference for the HP side overall, as indicated in Figure 5 and by the lack of a positive intercept or positive main effects of Time in the model.

As with the full sample, the LP event on Trial 9 influenced looking behavior for infants in both conditions. However, with this restricted sample, infants’ prior experience with the reliability of the target location moderated the effect of trial. For infants in the Deterministic condition, the effect of the Trial 9 LP event on Trial 10 looking behavior was relatively subtle, even though infants in this condition had never before seen the target appear on this side of the screen. This suggests that a single novel instance did not strongly shift infants’ expectations given their highly consistent global context. Infants in the Probabilistic condition, who had more variable prior experience with the target location, were more susceptible to trial-by-trial changes in the location of the target. As a group, they actually shifted to a preference for the LP side on the subsequent trial, even though the infants with the strongest preference for the LP side were excluded from this analysis.

## Second Low-Probability Set: Trials 13 and 14

The second LP trial set provides another opportunity to examine the length of the window that informs infants’ predictions. While the two groups of infants had quite different experiences with target predictability early in the experiment, all infants saw the same sequence of events from Trial 7 on. Thus, if infants’ anticipatory behavior is primarily governed by the most recent few trials, one would expect the two groups to have similarly strong expectations for the target location on Trial 13, the next LP trial, and to respond similarly on the subsequent trial. However, if global target predictability continues to influence looking behavior, infants in the Probabilistic condition (with their more variable prior experience) should again show stronger effects of the LP event on Trial 13 in their expectations on Trial 14, at least in the restricted sample.

**Figure 6:**
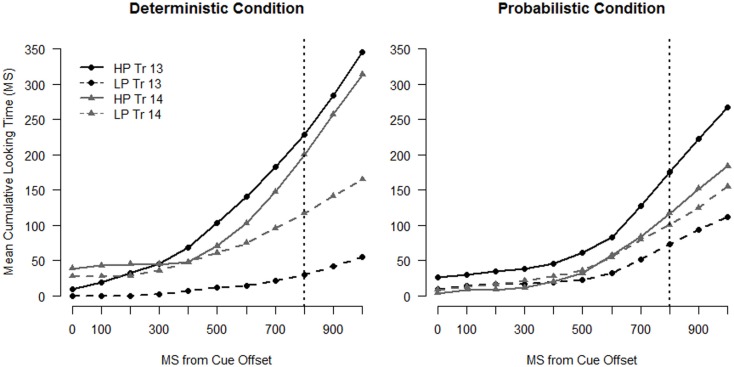
**Cumulative time spent looking to the high probability (HP) and low-probability (LP) location for infants in the Deterministic condition (left panel) and infants in the Probabilistic condition (right panel) for Trial 13 and Trial 14**. The dotted vertical line indicates target onset.

**Figure 7:**
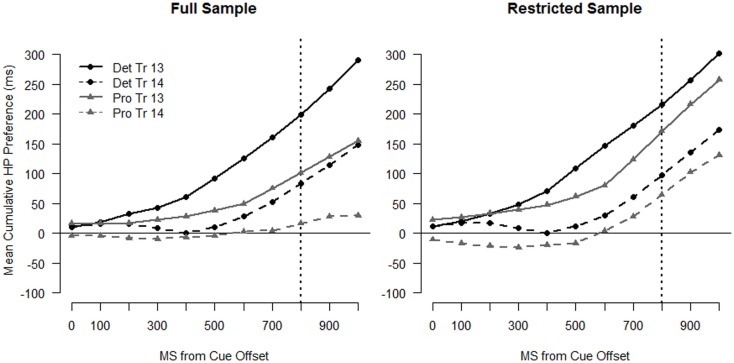
**Cumulative preference profiles for Trials 13 and 14**. The left panel depicts data from the full sample and the right panel depicts data from the restricted sample. Points above 0 indicate a preference for the HP location and points below 0 indicate a preference for the LP location. The dotted vertical line indicates target onset.

Looking behavior on Trials 13 and 14 was very similar for the two groups, both in the full sample and when the LP-predicting infants were excluded. The cumulative looks to the HP and LP locations are shown in Figure 6, and the HP location preference curves are shown in Figure 7. Model results for the two samples were the same, so we report statistics only from the full sample model (*N* = 24 in each condition, see Table A3 in Appendix).

The model revealed that infants in both conditions shifted their attention more to the LP side on Trial 14 than Trial 13. The Trial by Linear Time interaction was significant, and the negative coefficient indicates that the preference curves on Trial 14 are shallower than those on Trial 13 [χ^2^(1) = 35.53, *p* < 0.001]. The three-way interactions between Condition, Trial and Linear (and Quadratic) time were not significant, indicating that this second LP event had a similar influence on infants in both conditions.

The growth curve model also confirmed that by Trial 13 the two groups of infants had similar expectations about the target location. Neither the main effect nor interactions with Condition were significant (all *t* < 1). Both groups of infants anticipated the target at the HP location on Trial 13, as evidenced by the overall positive value of the preference curves and their positive slopes (the model intercept and the main effects Time were positive and had *t* > 2). That preference was maintained, if weakened, on Trial 14.

The results for the second LP set (Trial 13–14) are quite different than the results for the first LP set (Trial 9–10). The lack of Condition effects in the second set suggests that by mid-way through the experiment, the two groups were behaving similarly, despite divergent early experiences with the predictability of the target location. This is an interesting finding that reveals multiple factors influencing infants’ expectations in this task. First, there is a strong effect of immediate context, as revealed in the model by the Trial by Linear Time interaction. Second, infants’ looking behavior is also driven by experience over a larger temporal window. The similarity of the looking curves between conditions on Trials 13 and 14 suggests that infants in the two groups based their expectations on similar information. On the whole, by Trial 13 the two groups’ experiences were more similar than different, with only two of the first 12 trials (16%) differing between the two conditions (compared with Trial 9, at which point 25% of the trials had differed between conditions). By Trial 13, infants in the Probabilistic condition have begun to anticipate the target at the HP location and while they still show a decrement in looks to the HP location after the LP event at Trial 13, they, like infants in the Deterministic condition, maintained an overall preference for the HP location.

Finally, it is interesting to note that infants in the Probabilistic condition increased their preference for the HP location between Trials 9–10 and Trials 13–14. For these infants, the probability that the target will appear on the HP side is close to identical for these two sets of trials, whether that probability is calculated based on all previous trials or just the preceding six trials for each set. However, infants in the Probabilistic condition increased their anticipatory looks to the HP location and decreased their anticipatory looks to the LP location from Trial 9 to Trial 13. The only difference in experience with the sequence between these trials is more examples of the target appearing at the HP location. This accumulation of positive evidence for the HP side led infants to continue to increase their expectation that the target will appear there, even though statistically they did not gain any certainty.

## Discussion

The design of the current study enabled us to examine learning in real time. We measured infants’ predictions of the target location as they gained experience with a simple visual pattern. We found that infants initially exposed to a deterministic target location rapidly learned to predict the target at that location. Infants who were initially exposed to a probabilistic target location were much slower to begin predicting the likely location, though their rate of predicting the HP target location increased with experience. We also found that infants adjusted their expectations in real time. When the target appeared in an unlikely location, infants were more likely to anticipate the target at that same location on the subsequent trial than they were before.

### Learning from probabilistic input

The current study provides a framework within which to ask how infants represent probabilistic information. To date, the vast majority of studies investigating infant statistical learning have focused on how infants track deterministic structures (e.g., Haith et al., 1988; Aslin et al., 1998; Gómez and Maye, 2005; Kirkham et al., 2007; Smith and Yu, 2008). However, the domains that these laboratory studies are intended to model are not deterministic: the conditional probability between speech syllables within words is considerably less than 1.0, features within a category are not perfectly correlated, and words can be used in the absence of their referents. In one experiment that did explore probabilistic word learning, Vouloumanos and Werker (2009) showed objects to 18-month-old infants while presenting two different labels. One label was paired with the object more consistently than the other, and testing revealed that infants associated the more consistent label with the object. However, infants also kept track of the lower-probability pairings, as evidenced by competition between higher- and lower-probability object-label matches during the test session; it was not the case that infants simply “threw away” the noise.

Indeed, findings from several domains suggest that infants are highly attuned to the probabilistic structures in their environment. Infants are surprised by event outcomes that are statistically unlikely, such as when several red objects are randomly selected from a container in which white objects vastly outnumber red objects (Xu and Garcia, 2008; Téglás et al., 2011). Infants also are most likely to maintain attention to a visual sequence when the sequence is neither too predictable nor too unpredictable (Haith et al., 1969; Collins et al., 1972; Kidd et al., 2012) and attend more to language input that has learnable structure than structure that is unlearnable (Gerken et al., 2011). This work implies that infants readily attend to (and learn from) probabilistic input as long as it is not too irregular and contains some useable structure.

In the current study, the rate at which infants in the Probabilistic condition predicted the higher probability target location changed dramatically over the course of the experiment. In the early blocks, infants in the Probabilistic condition were equally likely to predict the target at the high and LP locations. In fact, the full sample of infants in the Probabilistic location did not reliably anticipate the target at the HP location until the very last block of trials. This raises the question of whether these infants learned anything about the target location during the first four blocks of the experiment. The trial-by-trial analysis provides some evidence that they did. Infants in the Probabilistic condition clearly increased their anticipations of the target at the HP location from the first to the second LP set (i.e., from Trial 9 to Trial 13), even though the *probability* that the target would appear on the HP side was the same for these two trials, 75%. The regression model for the second LP set indicated that infants in both conditions were anticipating the HP location at similar rates on Trials 13 and 14. These results suggest that that the accumulation of positive evidence is particularly important in the Probabilistic condition. Infants in this condition increased their predictions for the HP location even before the statistical structure of the input had changed. Frequency and conditional probability both contribute to infants’ acquisition of visual sequences (Marcovitch and Lewkowicz, 2009), and our data illuminate how frequency can boost expectations while probabilities are held relatively constant.

Inspection of individual data underscores the differences between the two conditions. While many infants in both conditions followed a response pattern similar to the group means, increasingly anticipating the target at the most likely location, some infants consistently predicted the target in the same location throughout the experiment. A subset of infants, primarily from the Probabilistic condition, did not appear to be sensitive to the probability structure of the materials and consistently anticipated the target at the *less* likely location. These LP-predicting infants raise interesting questions about the effects of variability. They suggest that the higher variability in the Probabilistic condition induced larger variation in learning outcomes relative to the Deterministic condition. When the LP-predicting infants were excluded, infants in both conditions anticipated the target at the HP location at similar rates. To some extent, this convergence is a necessary effect of removing the infants from the bottom of the distribution. However, we believe it is revealing that three-fourths of the sample of infants in the Probabilistic condition anticipated the HP location at such a high rate. Our data cannot tell us what precisely is driving the LP-predicting behavior in the subgroup of infants. It is possible that it reflects individual differences in the response to variability or the inhibition of prior responses. Future studies would benefit from collecting additional measures to help identify other correlates of this behavior. The variation we observed amongst infants in the Probabilistic condition suggests that studies investigating learning of probabilistic structures may need large sample sizes in order to gain sufficient power.

The extent to which learners’ behavior reflects the probability structure of the input is a question that has implications across domains. For example, some researchers have hypothesized that adults and children may differ in how they encode and use probabilistic linguistic structures, and these differences may play an important role in both language learning and language change (Hudson Kam and Newport, 2005, 2009; Wonnacott and Newport, 2005; Davis et al., 2011). If infants and children do not represent variability in language structures but rather encode only the most frequent structures, this selective encoding would allow young learners to shape the structure of language as they acquire it. Translating this hypothesis to the current study, the question is whether infants in the Probabilistic condition are *matching* (i.e., choosing each location at the rate at which it is rewarded) or *maximizing* (i.e., consistently choosing the most likely location, presumably to get the maximum expected reward). In the current study, we found that both local and global context influenced infants’ predictions, suggesting that the degree of probability matching behavior will depend on the window of time used to measure the behavior. Local variability in expectations complicates how one distinguishes matching and maximizing behaviors.

Even if one just examines the overall rate of predicting the HP location, the variability in behavior poses a challenge to determining whether infants are matching or maximizing. Maximizing behavior for infants in the Probabilistic condition would entail predicting the HP side at ceiling, which is unknown but presumably at or above the performance of the Deterministic condition. Matching, on the other hand, would entail predicting the HP side more frequently than the LP side (i.e., more often than chance) but less than ceiling. This leaves a very narrow range of possible scores to support probability matching. In a recent study also using a two-choice anticipatory eye movement paradigm, Davis et al. (2011) found that infants exposed to a probabilistic sequence in which the target appeared at the HP location 70% of the time predicted the HP location significantly less than a group exposed to a deterministic sequence. However, the infants exposed to the probabilistic sequence did not predict the HP location at a rate higher than chance, making it unclear whether they are representing the likelihood of the outcome.

Ultimately, arguments about how finely infants represent the statistics of the input must be made at the individual, rather than the group level. A group response rate at approximately the sequence probability (e.g., 75%) is not sufficient for demonstrating that individual infants are representing the probability as such, since the average could be derived from a bimodal distribution of individual response rates (for example, 100 and 50%). Indeed, this situation is analogous to the results from the Probabilistic condition in the current study, in which a minority of infants in the Probabilistic condition consistently predicted the LP side and the remaining infants predicted the HP side at a similar rate to the infants in the Deterministic condition. Supporting this view, recent work with adults suggesting that there are multiple possible mechanisms leading to probability matching (Gaissmaier and Schooler, 2008; Otto et al., 2011). Methods that enable analysis of individual performance are central to answering questions about probability matching.

### How do infants update representations of sequential input?

A primary goal of our study was to investigate not just learning outcomes, but how infants update their representations as they gather more information. The trial-by-trial analysis of the LP trial sets (Trials 9–10 and 13–14) revealed that infants include information across multiple time windows when forming their predictions. There was a significant effect of local context – the previous trial – on infants’ current predictions for the target location. After the first trial in the sets, on which the target appeared in the *less* likely location, both groups of infants increased their looking to that LP side. However, as one would expect from the results of the analysis across blocks, the global context also influenced the dynamics of infants’ expectations. Evidence for this larger window of influence comes from comparisons between the conditions as well as different time points within each condition.

Though both groups of infants made some shift in expectations during the first LP set, the change was stronger for the Probabilistic than the Deterministic group when the LP-predicting infants were excluded from the analysis. This result suggests that the greater variability experienced by infants in the Probabilistic group led them to shift their expectations more readily. In the second LP set, the gaze dynamics of the groups were more similar, both within and across trials. This suggests that while the first “LP” event did not have large immediate effects on the anticipatory behavior of infants in the Deterministic condition, it did influence their expectations more globally, making them more susceptible to later changes in target location.

The increase in flexibility of expectations with increased variability in experience ties in with other recent studies of infant pattern learning. In an experiment with 7-month-old infants, Kovács and Mehler (2009) used a similar design to teach infants that a verbal or visual cue predicted a target in a particular location. After nine trials of experience with this sequence (similar to our Deterministic condition), the target location switched to the opposite side and continued on that side for the next nine trials. Infants from bilingual homes switched their predictions, anticipating the target in the new location, while infants from monolingual homes did not. The authors argue that the larger amount of variability that infants experience in bilingual homes helps them to make the rule switch. Our current study shows that a lifetime of experience with multiple languages is not necessary in order to adapt to change: After experience with only one LP event, infants in the Deterministic group adjusted their predictions in the face of a second LP event (Trial 13) just as much as those in the Probabilistic group. As our study was designed to answer different questions, we do not know whether infants would have successfully switched their predictions if we had changed the pattern after Trial 13. Our participants were 5 months older than those in Kóvacs and Mehler’s study, and the developmental improvements in attentional control that occur during the first year of life (Colombo and Cheatham, 2006) no doubt contributed to the flexibility observed in the current study. Additionally, in adult reinforcement learning, large variability in reward leads to more exploratory behavior than a consistent reward with the same expected value, a phenomenon known as the payoff variability effect (Erev and Barron, 2005). It is possible that the effects of variability in the current experiment are also instances of this phenomenon.

Our finding that infants’ predictions are informed by a window of experience dominated by immediate context is compatible with classic learning models that update association strength based on the immediately preceding trial as well as all prior evidence, such as the Rescorla–Wagner model (Rescorla and Wagner, 1972) and simple recurrent networks (Elman, 1990). We did not find evidence, however, that changes to infants’ predictions were driven by the strength of the error signal generated by the unexpected event, as would be predicted by these models. If that were the case, infants in the Deterministic condition, for whom the first “LP” test set showed the target in a novel location, should have shown a larger shift in behavior than infants in the Probabilistic condition. However, our data represent a first step in investigating on-line learning processes in infants, and it would be premature to draw strong conclusions about learning mechanisms from them.

Finally, our results accord well with recent probability learning data from adults and non-human primates. Rhesus monkeys show rapid adaptation to changes in rate of reward, suggesting that their behavior is informed by a moving local window of reward (Sugrue et al., 2004). It is important to note, however, that choosing each item at the rate it was rewarded (probability matching) was the optimal behavior for Sugrue et al.’s task, while for our task maximizing would lead to the greatest opportunity for reward (i.e., the most time viewing the target animations). In a recent adult study, participants performed a reward prediction task with probabilistic cues under either a single task or dual task paradigm (Otto et al., 2011). Interestingly, decision-making models fit to the data suggested that participants under higher cognitive load used a longer window of trials to inform their current predictions. While it is impossible to know how our infant data compares with the adults’ performance, Otto et al.’s findings suggest that one source of variation in participants’ expectations may be their available cognitive resources, either due to task demands or individual differences. Ultimately, this variation may have consequences for the developmental trajectory of learning from probabilistic information.

Taken together, our findings reveal important information about infant expectancy learning. They suggest that the formation of expectations is very rapid when information is consistent, and that variable information influences expectations in many ways, including potentially highlighting individual differences in learning. One important goal for future work will be to test enough participants (perhaps with more trials as well) in order to compare how well different learning models fit both the group and individual participant data. For example, infants may vary in how strongly they weight the local and global contexts, with some infants displaying win-stay-lose-shift behavior and others an overall expectancy matching behavior or rule-learning behavior. The likelihood of all of these behaviors may be strongly influenced by the strength of the probabilities to be learned.

## Conclusion

Establishing learning outcomes at the group level is an important first step for understanding which cues infants are able to track and how cues trade off with one another. To understand learning *mechanisms*, in addition to outcomes, we must develop new methods that allow us to interrogate the learning process itself. The trial-by-trial analyses in our study offer an initial step in examining real time updating of infants’ expectations and revealed that behavior is influenced by information at multiple time spans. Understanding how infants build up representations of their environment will enable us to better identify differences in learning trajectories within and across groups, providing a powerful tool for studying learning and development.

## Conflict of Interest Statement

The authors declare that the research was conducted in the absence of any commercial or financial relationships that could be construed as a potential conflict of interest.

## Footnotes

^1^We would like to thank the reviewers for this suggestion.

## Appendix

**Table A1:** **Growth curve model results for Trials 9 and 10 for the full sample**.

	Variance	SD	
**RANDOM EFFECTS**			
Subject (intercept)	6.87	2.62	
Subject:trial	10.43	3.23	
Subject:linear time	99.93	10.0	
Subject:quadratic time	20.55	4.53	
Residual	4.62	2.15	

	**Estimate**	**SE**	***t* value**

**FIXED EFFECTS**			
Intercept	0.52	0.52	1.00
Condition	3.65	1.04	3.51
Trial	−0.28	0.70	0.40
Linear time	0.71	1.49	0.48
Quadratic time	−0.09	0.71	0.13
Condition:trial	0.89	1.40	0.64
Condition:linear time	9.31	2.99	3.12
Condition:quadratic time	3.02	1.41	2.14
Trial:linear time	−2.50	0.46	5.45
Trial:quadratic time	−1.21	0.46	2.65
Condition:trial:linear time	0.84	0.92	0.92
Condition:trial:quadratic time	1.04	0.91	1.14

*Deterministic condition *N* = 24, Probabilistic condition *N* = 22 (two infants had missing data for these trials)*.

*Both Condition and Trial were coded for main effects*.

**Table A2:** **Growth curve model results for Trials 9 and 10 for the sample of infants who were sensitive to the probability structure of the materials**.

	Variance	SD	
**RANDOM EFFECTS**			
Subject (intercept)	4.55	2.13	
Subject:trial	10.30	3.21	
Subject:linear time	82.53	9.08	
Subject:quadratic time	16.58	4.07	
Residual	4.88	2.21	

	**Estimate**	**SE**	***t* value**

**FIXED EFFECTS**			
Intercept	1.08	0.50	2.15
Condition	2.90	1.00	2.88
Trial	−0.61	0.74	0.82
Linear time	2.51	1.47	1.70
Quadratic time	0.71	0.70	1.02
Condition:trial	1.66	1.48	1.12
Condition:linear time	6.60	2.95	2.24
Condition:quadratic time	2.13	1.39	1.53
Trial:linear time	−3.05	0.50	6.10
Trial:quadratic time	−1.58	0.50	3.15
Condition:trial:linear time	2.18	1.00	2.18
Condition:trial:quadratic time	−0.23	1.00	0.23

*Deterministic condition *N* = 23, Probabilistic condition *N* = 17*.

*Both Condition and Trial were coded for main effects*.

**Table A3:** **Growth curve model results for Trials 13 and 14 for the full sample**.

	Variance	SD	
**RANDOM EFFECTS**			
Subject (intercept)	10.92	3.31	
Subject:trial	29.63	5.44	
Subject:linear time	111.34	10.55	
Subject:quadratic time	13.31	3.65	
Residual	7.76	2.79	

	**Estimate**	**SE**	***t* value**

**FIXED EFFECTS**			
Intercept	1.65	0.75	2.19
Condition	1.41	1.53	0.94
Trial	−1.92	1.16	1.65
Linear time	4.18	1.55	2.69
Quadratic time	1.56	0.50	2.59
Condition:trial	−0.42	2.33	0.18
Condition:linear time	3.40	3.10	1.10
Condition:quadratic time	1.03	1.21	0.85
Trial:linear time	−3.71	0.60	6.18
Trial:quadratic time	−0.17	0.59	0.28

*Deterministic condition *N* = 24, Probabilistic condition *N* = 24*.

*Both Condition and Trial were coded for main effects*.
